# Comparative Analysis of Rescue-In Vitro-Maturation (r-IVM) Outcomes in Women with Diminished Ovarian Reserve (DOR) Versus Normal Ovarian Reserve (NOR)

**DOI:** 10.3390/biomedicines13051084

**Published:** 2025-04-29

**Authors:** Mohd Faizal Ahmad, Nurul Yaqin Mohd Nor, Mohammad Mahmoud Mohammad Ramadneh, Nurul Izyani Roseli, Marjanu Hikmah Elias, Norazilah Mat Jin, Muhammad Azrai Abu, Saiful Effendi Syafruddin, Ani Amelia Zainuddin, Shah Shamsul Azhar, Nao Suzuki, Abdul Kadir Abdul Karim

**Affiliations:** 1 Advanced Reproductive Centre (ARC) HCTM UKM, Department of Obstetrics & Gynecology, Faculty of Medicine, National University of Malaysia, Jalan Yaacob Latiff, Bandar Tun Razak, Kuala Lumpur 56000, Malaysia; drmohdfaizal@ukm.edu.my (M.F.A.); yaqinahmed@gmail.com (N.Y.M.N.); ramaneh@gmail.com (M.M.M.R.); izyani.rosley@gmail.com (N.I.R.); azraiabu1983@gmail.com (M.A.A.); aniameliaz71@gmail.com (A.A.Z.); 2 Department of Obstetrics and Gynaecology, Kuliyyah of Medicine International Islamic University Malaysia, Kuantan 25200, Pahang, Malaysia; 3 Department of Obstetrics and Gynaecology, Arrawdha General Hospital, Dammam 32233, Saudi Arabia; 4 Faculty of Medicine & Health Sciences, Universiti Sains Islam Malaysia, Nilai 71800, Negeri Sembilan, Malaysia; marjanuhikmah@usim.edu.my; 5 Department of Obstetrics & Gynecology, Faculty of Medicine, Universiti Teknologi MARA, Sungai Buloh Campus, Selangor Branch, Jalan Hospital, Sungai Buloh 47000, Selangor, Malaysia; drnorazilah@yahoo.com; 6 Medical Molecular Biology Institute, National University of Malaysia, Jalan Yaacob Latiff, Bandar Tun Razak, Kuala Lumpur 56000, Malaysia; effendisy@ppukm.ukm.edu.my; 7 Department of Public Health, Faculty of Medicine, Universiti Kebangsaan Malaysia, Cheras, Kuala Lumpur 56000, Malaysia; drsham@ppukm.ukm.edu.my; 8 Department of Obstetrics & Gynecology, St Marianna School of Medicine, Kawasaki 216-0015, Kanagawa, Japan; nao@marianna-u.ac.jp

**Keywords:** rescue-IVM, in vitro maturation, IVF-ICSI, diminished ovarian reserve, oocyte maturation rate

## Abstract

**Background/Objectives:** Diminished ovarian reserve (DOR) poses significant challenges in the reproductive field, resulting in fewer mature and more low-quality eggs. **Methods:** We studied r-IVM in addition to standard in vitro fertilization (IVF) and compared the embryological outcomes between both DOR and NOR women. **Results:** We recruited 90 women (45 NOR; 45 DOR) with a younger age seen in NOR (35.2 vs. 36.5 years old) women. Otherwise, DOR women had lower levels of AMH and AFC, thus fewer retrieved follicles and collected oocytes. Most of the group presented with primary subfertility, with 55.6% in the NOR group diagnosed with polycystic ovary syndrome (PCOS), while 37.8% in the DOR group presented with aging and cancer survivorship issues. Most women in the NOR group used hCG as a trigger (82.2%), while 17.8% of the DOR group opted for a decapeptide. A total of 719 oocytes were retrieved, with 72.3% of eggs being mature in the NOR group compared to 64.9% in the DOR group. Following r-IVM, 47.69% of NOR eggs were matured compared to 60% in DOR eggs. The fertilization rates (FRs) following r-IVM were higher in the DOR group (66.7% vs. 37.8%). Overall, higher numbers and quality of D3 embryos were seen in the DOR group. Our analysis revealed that the trigger type, hCG, was the only significant factor linked to successful oocyte maturation rates. **Conclusions:** Our study suggests that r-IVM may enhance outcomes for women with DOR, including better egg maturity, FR, and embryo quality than NOR women.

## 1. Introduction

Diminished ovarian reserve (DOR) poses a significant challenge in assisted reproductive technology due to the complexity of its management. Women with DOR often experience suboptimal outcomes with conventional controlled ovarian hyperstimulation (COH) protocols, resulting in an insufficient number of mature oocytes and a high proportion of poor-quality oocytes [1,2]. This condition is further complicated by age or other factors that lead to low anti-Mullerian hormone (AMH) levels (0.5–1.1 ng/mL) and a reduced antral follicle count (AFC < 5). Commonly observed through ultrasonography (USG), less than three oocytes are retrieved following the conventional stimulation protocol [3]. These characteristics are indicative of DOR as defined by the Bologna criteria established during a consensus meeting of the European Society of Human Reproduction and Embryology (ESHRE) working group on the POR definition held in 2011 [4]. The predominant causes of DOR in women include age-related physiological decline, chronic endometriosis, and the adverse effects of radiotherapy or chemotherapy in cancer survivors, all of which negatively affect folliculogenesis and reduce the overall oocyte reserve [5]. Compared to with women with usual ovarian reserve (NOR), relying on a large pool of high-quality oocytes, most women with DOR require alternative strategies to optimize their in vitro fertilization (IVF) outcomes [6]. Various adjunct therapies have emerged, including growth hormone supplementation, laboratory enhancements such as platelet-rich plasma, and in vitro maturation (IVM) [7,8,9,10]. The latter was initially developed to mitigate ovarian hyperstimulation syndrome (OHSS) in women with polycystic ovarian syndrome (PCOS) and has undergone substantial advancements [11,12].

Currently, the maturation rate of human oocytes in vitro is reported to be between 30% and 50%, which is markedly lower than the 80% observed in animal models [13,14]. IVM applications have diversified to encompass fertility preservation for patients undergoing cancer treatment, as well as the potential for oocyte and embryo cryopreservation [15]. However, most evidence has highlighted the suboptimal results of IVF within standard cycles in the DOR group. At least 20% to 30% of retrieved oocytes remain immature after standard hCG priming in COH-IVF cycles and are typically discarded [16,17]. Therefore, in the DOR group where every oocyte counts, these immature oocytes can be salvaged through IVM culture to improve their maturation outcomes [18]. IVM has been proposed as the standard, with or without gonadotrophin stimulation, and oocyte pick-up (OPU) is performed without a trigger agent [19]. Thus, complete maturation will be achieved in vitro with or without capacitation protocol IVM (CAPA IVM) [20]. In the practice of IVM culture, following standard COH with a trigger agent is considered a non-standard practice known as hCG–primed IVM or rescue-IVM (r-IVM). This protocol involves culturing the immature oocytes obtained during standard COH-IVF cycles. The culture technique enhances the developmental competency of cumulus–oocyte complexes (COCs) by promoting their maturation from the germinal vesicle (GV) and meiosis I (MI) stages and progression into the meiosis II (MII) stage [20,21]. Incorporating r-IVM strategies, such as hCG priming combined with mild ovarian stimulation, has been shown to improve outcomes significantly and can be suggested as an additional approach for women with DOR [22]. In the DOR group, the asynchronous recruitment of follicles leads to a higher proportion of GVs and metaphase I (MI) oocytes than in women with NOR [23,24]. As a result, the oocyte maturation rate is higher in the NOR group. Current evidence suggests that r-IVM as an additional strategy for women with DOR offers promising outcomes [21]. The evidence indicates improvements in oocyte maturation, fertilization, and embryo quality [1,15,18]. However, the inconsistent implementation of r-IVM in clinical practice has created a research gap on its role in this group. Therefore, this study aims to evaluate and compare the overall outcomes of r-IVM among women with DOR and NOR within our center. We seek to establish r-IVM as an optimal strategy for improving outcomes for women with DOR undergoing standard COH-IVF cycles. We also determine the factors influencing oocyte maturation rates following r-IVM and their subsequent impact on embryological outcomes.

## 2. Materials and Methods

### 2.1. Study Design

**Figure 1:**
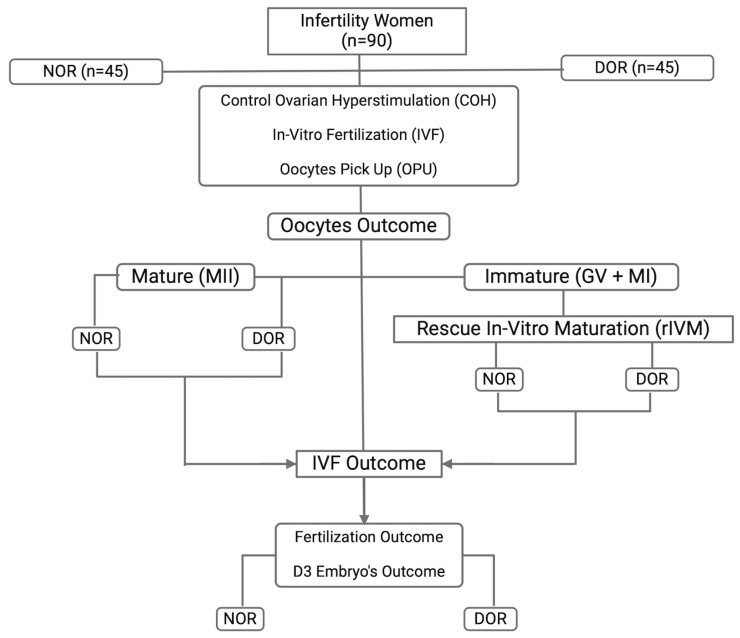
The study flow: group recruitment and IVF outcome.

This retrospective study was conducted from July 2021 to July 2022 at the Advanced Reproductive Center, Hospital Canselor Tuanku Mukhriz (HCTM) UKM, located in Cheras, Kuala Lumpur, Malaysia. Prior to recruitment, approval was obtained from the Human Ethical Research Committee (JEP-2023-360 - and JEP-2022-187), Faculty of Medicine, National University of Malaysia, Cheras, Kuala Lumpur, Malaysia. Informed and verbal consent was obtained from participants. Based on the Bologna criteria, participants were classified as either having DOR or NOR. The classification was determined by evaluating AMH levels and AFC. Specifically, DOR was identified in individuals with AMH levels below 1.2 ng/mL and an AFC of less than five as established through initial ultrasound assessments. All participants underwent controlled ovarian hyperstimulation (COH) for in vitro fertilization (IVF) using an agonist protocol. The selection of stimulation agents, whether standard or minimal, was determined according to clinician preference and the applicable local protocols (Figure 1).

### 2.2. COS, r-IVM, and IVF Protocols

**Figure 2:**
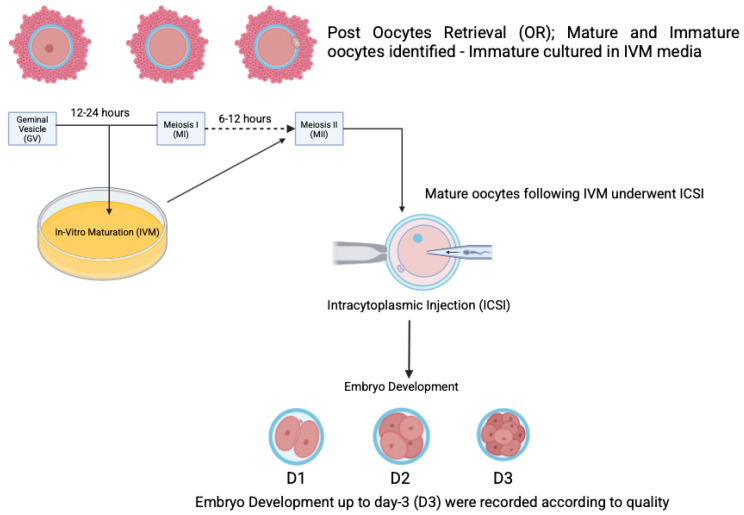
The rescue-IVM (r-IVM) procedure performed after obtaining immature oocytes.

**Figure 3:**
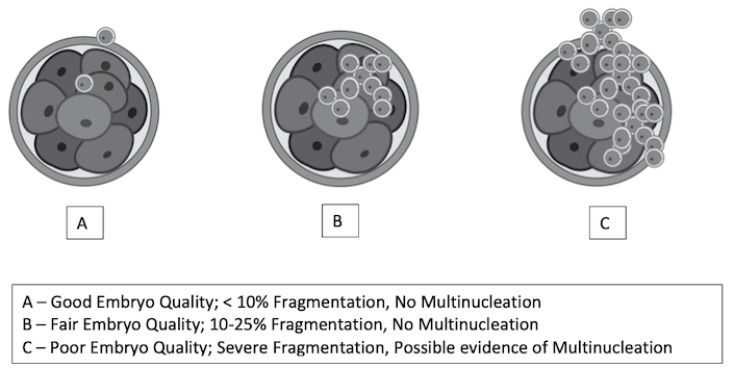
Embryo quality (EQ) grading based on fragmentation.

The standard stimulation protocol involved a combination of gonadotropins, specifically follitropin alfa (Gonaf F^®^, Merck Darmstadt, Germany) and highly purified menotrophin (Humog^®^ BSV, Mumbai, India), administered at a dosage of 225 IU daily for a duration of 1 to 10 days. An antagonist, Cetrotide^®^ (Merck, Germany), was introduced once the follicles reached 10 mm to 12 mm in size. For the minimal stimulation protocol, an oral agent, either an aromatase inhibitor or clomiphene citrate, was used during the first five days, along with highly purified menotrophin (Humog^®^ BSV, Mumbai, India) at 225 IU on alternating days (specifically on days 3, 5, and 9) of ovarian stimulation. An antagonist (Asporelix^®^, BSV, Mumbai, India) was administered when the follicles reached 10 mm to 12 mm in size. When two or more dominant follicles reached a diameter of 18 mm, an ovulation trigger was provided. The options for this trigger included Ovidrel^®^ (Merck, Darmstadt, Germany), Hucog^®^ (BSV, Mumbai, India), Decapeptyl^®^ (Ferring, Saint-Prex, Switzerland), or a dual trigger comprising any two of the aforementioned variants. The OPU procedure was scheduled 36 h post-trigger administration. All participants underwent OPU as part of the standard protocol, during which the COCs were evaluated for oocyte maturity. All mature oocytes (MII) were subjected to intracytoplasmic sperm injection (ICSI) at least 4 h after administering the hCG/LH trigger. By contrast, the immature oocytes (MI, GV) were cultured in Kitazato^®^ (Tokyo, Japan) IVM medium for periods of up to 48 h (Figure 2). The maturation process was closely monitored at intervals of 12, 24, and 48 h, with ICSI performed upon achieving metaphase II (MII). Any oocytes that remained immature after 48 h of culture were discarded. Embryological outcomes were subsequently evaluated, with the primary outcome being the oocyte maturation rate (OMR), defined as the ratio of MII oocytes to the immature oocytes cultured. Secondary outcomes included the fertilization rate (FR), calculated as the percentage of micro-injected MII oocytes that developed into two pronuclei (2PN). The cleavage rate was defined as the total number of day 3 (D3) embryos relative to the total number of fertilized oocytes. The overall quality of D3 embryos was assessed according to the ESHRE Istanbul Consensus. Specifically, good embryo quality was characterized by less than 10% fragmentation and the absence of multinucleation. Fair embryo quality was characterized by 10% to 25% fragmentation without multinucleation. Conversely, poor embryo quality was indicated by severe fragmentation, potentially accompanied by multinucleation (Figure 3). In the present group, only D3 embryos were cultured, cryopreserved, or transferred according to the IVM protocol at the clinician’s discretion.

### 2.3. Statistical Analysis

All collected data were presented according to their distribution, using mean ± standard deviation (SD) for normally distributed data and median ± interquartile range (IQR) for skewed data. Statistical comparisons were conducted using a two-tailed Student *t*-test, with statistical analysis performed using SPSS software version 29.0 (IBM^®^ SPSS^®^ software). A *p*-value of less than 0.05 was regarded as statistically significant.

## 3. Results

A total of 90 women were recruited for this study, with 45 classified as having NOR and 45 categorized as DOR. The mean age of participants was comparable between the two groups, with the NOR group averaging 36.5 years (±4.24) and the DOR group averaging 35.2 years (±4.88) (*p* = 0.68). In addition, the duration of fertility issues was similar across both groups, exhibiting no significant difference (*p* = 0.77). The DOR group presented with significantly lower levels of AMH and AFC, as well as a non-significantly decreased number of follicles aspirated per cycle, resulting in a lower follicular oocyte index. Consequently, women in the DOR group yielded fewer oocytes than those in the NOR group (*p* = 0.035). A majority of the participants were diagnosed with primary subfertility, with the NOR group comprising 55.6% and the DOR group consisting of 37.8%. The leading cause of subfertility among the NOR group was polycystic ovary syndrome (PCOS) (17.8%), whereas aging and cancer survivorship factors predominated in the DOR group (26.7%). In terms of trigger agents, the majority of women in the NOR group used human chorionic gonadotropin (hCG) (82.2%), whereas only eight women (17.8%) opted for a decapeptide. Conversely, over half of the DOR group followed the clinician’s recommendation regarding the use of an hCG trigger. A total of 714 oocytes were retrieved from the NOR group, compared to just 77 from the DOR participants (Table 1).

On average, each woman in the NOR group yielded at least 16 oocytes, with 21.9% being immature (GV and MI), 5.3% classified as abnormal, and 72.3% being mature (MII). In comparison, each woman in the DOR group yielded at least two oocytes, with 64.9% being mature (MII), 25.9% classified as immature (GV and MI), and 9.1% being abnormal and subsequently discarded. The in vivo maturation rate was comparable between the DOR and NOR groups (*p* = 0.074). Regarding immature oocytes, after 24 h of IVM culture, 47.69% and 60% matured to MII in the NOR and DOR groups, respectively (*p* = 0.006) (Table 2). In vivo fertilization outcomes were similar between the NOR and DOR groups (*p* = 0.78) in terms of FR. However, post-IVM fertilization revealed a significantly higher rate in the DOR group (66.7%) than in the NOR group (37.8%) (Table 3). Furthermore, at least 39.3% and 77.8% of day 3 embryos were significantly obtained from the NOR and DOR groups, respectively, following rIVM (*p* = 0.042). Women with DOR produced fair- to good-quality embryos significantly more often than those in the NOR group post-rIVM (*p* = 0.033) (Table 4). An analysis of factors influencing OMR across all participants, including age, infertility factors, ovarian reserve classification, and trigger type, was conducted (Table 5). The type of trigger administered emerged as the sole factor with a significant association with successful OMR, with hCG being favored (*p* = 0.027) and an adjusted odds ratio of 2.021 (95% CI 1.085–3.766) (Table 6). Finally, a number-needed-to-treat (NTT) analysis for IVM across both groups revealed a substantially higher NNT for the NOR group at 1:909 than the DOR group at 1:169 (Table 7).

## 4. Discussion

IVM is not a recent development; it originated over a century ago and has progressed from initial applications in animal models to those involving humans. Despite this long history, the clinical implementation of IVM remains limited, primarily due to inconsistent outcomes and the variety of protocols developed internationally [19,20,25]. Our fundamental understanding of this procedure has been hindered by different stimulation methods and culture media, which may or may not include triggering agents. In addition, the clinical indications for IVM are quite narrow, with the majority of evidence focusing on its application in hyperresponsive women to mitigate the risk of ovarian hyperstimulation syndrome (OHSS) [11,12]. Consequently, most clinical data pertain to women with a high ovarian reserve, particularly those diagnosed with polycystic ovary syndrome (PCOS). Many of the studies published to date have employed standard IVM techniques, typically performed without a triggering agent, and have resulted in oocyte retrieval (OPU) from small-diameter follicles. Recently, a two-step IVM approach known as CAPA-IVM has been introduced to improve maturation rates and outcomes [26]. As highlighted in the latest review conducted by our team, the indications for IVM have expanded to include women undergoing oncofertility treatments and, notably, those classified within the DOR group [20]. A significant challenge in managing women with DOR is obtaining mature oocytes in a single stimulation cycle. Despite using various adjuvants, the women with DOR in this study yielded low numbers of oocytes, with quality and maturation remaining suboptimal. Age is often correlated with ovarian reserve, and most women in the DOR group are often over 40 years old. As a result, many micromolecular environment abnormalities are attributed to age factors rather than ovarian reserve [27]. By contrast, although our DOR women were older, both groups were less than 40. Therefore, many abnormalities in the micromolecular environment are primarily attributable to age factors rather than to ovarian reserve itself. However, in our study, DOR and NOR groups consisted of women under 40 years old.

Consequently, our findings were limited to this younger group with DOR, providing a comparison to the standard age of the NOR group. Age-related factors were not linked to pathology and did not compromise the microenvironment; thus, favorable outcomes were observed following rescue-IVM (r-IVM) within our DOR group. As anticipated, immature oocytes are generally discarded in the NOR group because many mature oocytes remain available for ICSI. Our investigation revealed that at least 15% to 30% of oocytes from the NOR group were immature, in contrast to at least 30% to 40% in the DOR group. Furthermore, our analysis demonstrated a higher percentage of mature oocytes (MII) and a lower incidence of abnormal oocytes in the NOR group than in the DOR group along with favorable IVM results within the NOR group. Introduction of the r-IVM protocol in our study aims to enhance oocyte maturation following standard ovarian stimulation in women with DOR. The term “rescue” was used because this approach is considered a non-standard form of IVM, where OPU occurs after standard stimulation with adequately sized follicles and is preceded by an hCG or LH trigger. Contrary to standard IVM, r-IVM is not widely implemented globally [20,25]. Previous studies emphasize that using a trigger agent with dominant-size follicles promotes the premature resumption of meiosis, leading to poor oocyte maturation in the IVM cycle [26,28]. However, we discovered that r-IVM is a suitable strategy for achieving excellent OMR in our women with DOR. More than 50% OMR was recorded for the DOR group compared to 47.4% in the NOR group, thereby increasing the use of oocytes for ICSI. Our results align with the established literature, suggesting that r-IVM can enhance the maturation of immature oocytes for subsequent fertilization [21,29]. The evidence suggests that the dynamic environment surrounding oocytes differs significantly between women with DOR and those with NOR. In the DOR group, granulosa cell receptors and their associated maturation regulatory mechanisms may exhibit dysfunction in vivo [30,31]. However, they appear to be restored in vitro through the IVM medium. This reversal is likely attributable to previously suboptimal environments. Conversely, in the NOR group, the overall defective maturation mechanisms are likely permanent, often due to mutations, and are typically irreversible [32,33]. Our findings corroborate this observation as our OMR was significantly higher in the DOR group than in the NOR group. Most of our group used hCG as a trigger agent, which is considered a standard trigger in most IVF cycles globally [34]. However, rising evidence supporting the use of dual triggers for enhanced oocyte quality leads to a higher uptake of this strategy among women with DOR in our group [35]. Contrary to standard IVM, r-IVM requires an LH surge via triggering to ensure that the resumption of meiosis, which may not be achievable in vivo, can be rescued in vitro [36,37]. Previous studies on r-IVM have similarly exhibited this phenomenon [18,38]. Our study demonstrated that hCG significantly influences oocyte maturation outcomes. By contrast, additional factors, such as age, AMH levels—comparing DOR to NOR, and body mass index (BMI), did not appear to impact oocyte maturation within our group.

In our investigation of embryological outcomes, we observed that fertilization and embryo development in the in vivo group were comparable. Conversely, r-IVM in women with DOR resulted in superior FRs and enhanced embryo development. Notably, the proportion of good- to fair-quality D3 embryos was significantly elevated within our DOR group subjected to r-IVM. Theoretically, after oocyte maturation and ICSI, the subsequent processes of fertilization and embryo development are independent and primarily governed by the genetic material of the oocyte and sperm [39,40]. Consequently, our objective with r-IVM is to generate high-quality mature oocytes that can successfully advance to the embryo stage. Currently, our IVM protocol is classified as experimental within our institution; thus, embryos have been cryopreserved at the D3 stage instead of progressing to the blastocyst stage. As established in the literature, NTT analysis quantitatively assesses the absolute impact of medical interventions, determining their beneficial or detrimental effects [41]. Our group conducted the NTT analysis from a practical perspective, revealing that the NTT for r-IVM was lower for the DOR group than the NOR group. Our findings suggest that r-IVM is more cost-effective for women with DOR than those in the NOR group. Thus, it is an effective clinical strategy for the DOR group in our center.

Nevertheless, our study is limited by a small sample size and single-center experience. We acknowledge this limitation. Thus, future studies, preferably in a multicenter setting, should be proposed. In addition, our embryological outcome is confined to only the D3 embryo, with no data on embryo transfer outcome, particularly pregnancy outcomes. To date, all our r-IVM groups opted for frozen embryo transfer as the IVM culture protocol is up to 48 h prior to ICSI; thus, we do not report any pregnancy outcomes for this group at this time. Furthermore, per the manufacturer’s protocol, D3 embryos are recommended for our IVM media composition rather than D5 embryos. Therefore, we aimed only for D3 embryos to be frozen. Therefore, in future research, we aim to evaluate the outcomes of embryo transfer and pregnancy to consolidate our current findings.

## 5. Conclusions

In conclusion, our study revealed that r-IVM may offer favorable outcomes for women with diminished ovarian reserve (DOR), including higher oocyte maturity rates (OMR) and fertilization rates (FR) and enhanced embryo quality compared to women with normal ovarian reserve (NOR). Our findings contribute to a better understanding of the potential benefits of r-IVM in this population. The findings provide valuable clinical insights into r-IVM among women with DOR and support existing evidence on the efficacy of r-IVM among women with DOR. DOR should be considered a new indication for offering IVM, particularly r-IVM, as adjuncts in these challenging cases. Moreover, r-IVM should be implemented as part of clinical practice once sufficient evidence is gathered to consolidate its overall effectiveness among women with DOR. Further collaboration with multiple centers is essential to establish a specific consortium to identify solid outcomes of r-IVM in DOR for future implementation.

## Figures and Tables

**Table 1:** Baseline characteristics of both groups (n = 90).

Clinical Characteristic	NOR (Mean, SD)	DOR (Mean, SD)	*p*-Value
No of cycle	45	45	-
Age	35.2 (±4.88)	36.5 (±4.24)	0.68
Duration of infertility	6.8 (±4.5)	7.0 (±4.8)	0.77
AMH (ng/mL)	2.47 (±2.3)	1.08 (±0.5)	0.035 *
AFC	13.3 (±8.1)	2.4 (±1.06)	
No of follicle aspirated	932 (11, ±5)	109 (2, ±1.5)	
Retrieved oocyte number	714 (8, ±3)	77 (2, ±1)	
Follicular oocyte Index (FOI)	76.6% (±2.8)	70.6% (±2.1)	0.54
Causes of Infertility	NOR n, (%)	DOR n, (%)	*p*-Value
Primary	25 (55.6)	17 (37.8)	0.048 *
Secondary	3 (6.7)	1 (2.2)	0.38
Male factor	3 (6.7)	2 (4.4)	0.91
PCOS	8 (17.8)	0 (0)	0.002 *
Endometriosis	1 (2.2)	9 (20.0)	0.037 *
Tubal factor	3 (6.7)	1 (2.2)	0.16
Unexplained	2 (4.4)	3 (6.7)	0.48
Others (Age, Oncofertility)	0 (0)	12 (26.7)	0.001 *
Trigger Agent	NOR n, (%)	DOR n, (%)	
hCG	37 (82.2)	30 (66.7)	-
Decapeptide	8 (17.8)	0	
Dual trigger	0	15 (33.3)	

* Independent *t*-test.

**Table 2:** Maturation outcomes.

Oocytes Outcome	NOR (n:45) n(%)	DOR (n:45) n(%)	*p*-Value
No of oocyte retrieved	714 (100)	77 (100)	-
No of oocyte per patient (mean)	15.8	1.71	
No of MII day 0	516 (72.3)	50 (64.9)	
No of MI day 0	99 (13.9)	17 (22)	
No of GV day 0	57 (7.98)	3 (3.9)	
No of abnormal oocytes	38 (5.3)	7 (9.1)	
No of matured oocytes in vivo (MII day 1)	73 (46.8)	12 (60)	
No of matured oocyte (MII day 2)	1 (0.6)	0	
No of matured oocytes In vitro (IVM)	74 (100)	12 (100)	
In vivo maturity rate	72.3%	64.94%	0.074
In vitro maturation rate	47.4%	60%	0.006 *

* Independent *t*-test.

**Table 3:** Fertilization outcomes.

Fertilization Outcome	In Vivo Group		*p*-Value	In Vitro Group		*p*-Value
	NOR n (%)	DOR n (%)		NOR n (%)	DOR n (%)	
No of ICSI	516 (100)	50 (100)	*p* = 0.78	74 (100)	12 (100)	*p* = 0.042 #
No of fertilization (2PN)	282 (54.7)	25 (50)		28 (37.8)	9 (66.7)	
Fertilization rate	54.7%	50%		37.8%	66.7%	
Day 3 embryo	166 (58.9)	12 (48)		11 (39.3)	7 (77.8)	

# Mann–Whitney test.

**Table 4:** Outcomes of D3 embryo quality.

Embryo Quality Outcome	In Vivo Group		*p* Value	In Vitro Group		*p*-Value
	NOR	DOR		NOR	DOR	
Good	101 (60.8)	6 (50.0)	*p* = 0.76	1 (9)	1 (14.3)	*p* = 0.033 #
Fair	34 (20.5)	4 (33.3)		3 (27.3)	5 (71.4)	
Poor	31 (18.7)	2 (16.7)		7 (63.6)	1 (14.3)	

# Mann–Whitney test.

**Table 5:** Factors associated with the oocyte maturation rate (OMR).

Successful Oocyte Maturation	Simple Logistic Regression	
	OR (95% CI)	*p*-Value
Age		
<35 years 35–40 years >45 years	1 1.074 (0.588–1.964) 1.388 (0.672–2.865)	- 0.816 0.375
Factor of infertility		
Unexplained Single factor Two factors	1 0.6 (0.229–1.571) 0.269 (0.051–1.42)	0.298 0.122
Ovarian reserve (ng/mL)		
Normal ovarian reserve Diminished ovarian reserve	1 1.875 (0.880–3.995)	0.104
Type of trigger		
Dual trigger Decapeptide hCG	1 1.520 (0.348–6.644) 2.190 (1.197–4.007)	0.578 0.011 ^

^ OR: Odds ratio.

**Table 6:** Factors associated with the oocyte maturation rate (OMR).

Successful Oocyte Maturation	Multiple Logistic Regression	
	aOR (95% CI)	*p*-Value
Age <35 years 35–40 years >45 years	1 1.054 (0.560–1.983) 1.059 (0.482–2.323)	- 0.870 0.887
Factor of infertility Unexplained Single factor Two factors	1 0.713 (0.264–1.923) 0.306 (0.055–1.696)	0.504 0.175
Ovarian reserve (ng/mL) Normal ovarian reserve Diminished ovarian reserve	1 1.845 (0.805–4.229)	0.148
Type of trigger Dual trigger Decapeptide hCG	1 1.437 (0.318–6.489) 2.021 (1.085–3.766)	0.637 0.027 ^

^ aOR: adjusted odds ratio.

**Table 7:** The number needed to treat for IVM (NNT).

Group	In Vivo (MII)		In Vitro (MII)		NNT
	No of Oocyte	No of Embryo	No of Oocyte	No of Embryo	
NOR	516	166	74	11	1:909
DOR	50	12	12	7	1:169

## Data Availability

Data are contained within the article.

## Abbreviations

The following abbreviations are used in this manuscript:

ART	Artificial Reproductive Technique
cAMP	Cyclic Adenosine Monophosphate
COC	Cumulus-oocyte Complexes
DOR	Diminished Ovarian Reserve
FSH	Follicular Stimulating Hormone
GnRH	Gonadotrophin Releasing Hormone
GV	Germinal Vesicle
GVBD	Germinal Vesicle Breakdown
hCG	Human Chorionic Gonadotrophin
ICSI	Intra Cytoplasmic Sperm Injection
IVM	In Vitro Maturation
IQR	Interquartile Range
LH	Luteinizing Hormone
M1	Meiosis 1
MII	Meiosis II
OHSS	Ovarian Hyperstimulation Syndrome
OMR	Oocyte Maturation Rate
OTO-IVM	Ovarian Tissue Oocyte In Vitro Maturation
PB1	First Polar Body
PCOS	Polycystic Ovarian Syndrome
rIVM	Rescue IVM
2PN	2 Pronuclei
